# The clinical diversity and molecular etiology in 46, XY disorders of sex development patients without uterus

**DOI:** 10.1186/s13023-025-03719-y

**Published:** 2025-04-17

**Authors:** Leilei Ding, Min Luo, Shan Deng, Duoduo Zhang, Qinjie Tian

**Affiliations:** 1 Department of Obstetrics and Gynecology, Peking Union Medical College, National Clinical Research Center for Obstetric & Gynecologic Diseases, Peking Union Medical College Hospital, Chinese Academy of Medical Sciences, Beijing, 100730 China; 2 Center for Rare Diseases Research, Chinese Academy of Medical Sciences, Beijing, 100730 China

**Keywords:** 46, XY DSDs, Without uterus, WES, Reverse phenotyping, Molecular etiology

## Abstract

**Background:**

Disorders of sexual development (DSDs) are a group of rare conditions with a discordance of chromosomal, gonadal, or phenotypic features of the internal and/or external genitalia, which accounts for 0.5% of the population. The precise diagnosis of 46, XY DSDs without uterus is often obscure because of the similar clinical manifestations. Reverse phenotyping based on specific genetic variants helps to identify the cause of these diseases and reduces misdiagnosis caused by limitations in serum tests and imaging.

**Methods:**

Patients with 46, XY DSDs without uterus were enrolled from the Department of Obstetrics and Gynecology, Reproductive Endocrinology and Infertility Center of the Peking Union Medical College Hospital between 2022 and 2023. Comprehensive clinical data, including the social gender, chief complaint, physical examination results and laboratory tests related to sexual development, and surgical information were collected from medical records. Whole exome sequencing (WES) was performed on all patients and the etiological diagnoses were made based on the results. Targeted Sanger sequencing for the candidate gene was performed in the parents.

**Results:**

A total of twenty-one patients with 46, XY DSDs without uterus were included. Twenty-two variants from six genes associated with sex development were identified, including 14 recurrent variants and 8 novel variants. Based on the ACMG guidelines, 17 variants were classified as pathogenic (P) or likely pathogenic (LP), and 5 were defined as variants of uncertain significance (VUS). The genes *LH/HCG receptor (LHCGR)* (2/22), *CYP17A1* (4/22), *SRD5A2* (3/22), and *AR* (10/22) were involved in steroid hormone synthesis and androgen receptor action, while *NR5A1*(2/22) was associated with gonadal development. Furthermore, a *DHX37* variant instead of an *AR* variant was identified in a patient clinically diagnosed with complete androgen insensitivity syndrome. Trio-WES revealed three de novo variants.

**Conclusion:**

This study identified several novel variants broadening the mutation spectrum of 46, XY DSD without uterus. The etiology of 46, XY DSDs is complex. Reverse phenotyping helps differentiate the abnormalities and explore the molecular etiology more accurately.

**Supplementary Information:**

The online version contains supplementary material available at 10.1186/s13023-025-03719-y.

## Background

Disorders of sex development (DSDs) are a group of congenital developmental abnormalities characterized by atypical chromosomal, gonadal, or phenotypic features of the internal and/or external genitalia, exhibiting high heterogeneity in clinical manifestations and genetics. The prevalence of DSDs is about 1 in 4500 to 5000 newborns, accounting for 0.5% of the population [1]. According to the consensus of the 2006 Chicago conference, DSDs are divided into 46, XX DSD, 46, XY DSD, and sex chromosome DSD [2], a classification widely recognized in clinical practice. The global incidence of DSDs in 46, XY individuals is estimated at 1 case per 20,000 births [3], varying by region due to differences in the frequency of pathogenic variants.

The differentiation and development of the gonads is a complex process governed by the combination of genetic networks and hormonal signaling. The 46, XY DSDs involve multiple etiologies, including disorders of testicular development and androgen synthesis or action, leading to impaired virilization [4]. In 46, XY individuals, if the testes develop, functional testes secrete AMH to inhibit the development of Mullerian-derived structures (uterus, fallopian tubes, and upper vagina), leading to the absence of a uterus. Ultrasound (US) is the primary method for examining the presence of gonads and Mullerian-derived structures. Clinically, pelvic US is the initial diagnostic step for 46XY DSDs due to its convenience and cost-effectiveness [5]. 46XY DSDs are classified into two categories based on the presence or absence of the uterus on ultrasound.

The clinical phenotypes of 46, XY DSDs without a uterus are similar and complex, making it difficult to differentiate the underlying causes and identify the disease-causing genes. Clinical, chromosomal, and hormonal assessments may provide some but not enough evidence to make the etiological diagnosis. With the global development of precision medicine initiatives, genomic data significantly enhances our understanding of complex diseases. Reverse phenotyping, where phenotypes are refined based on genetic data, may be a promising new approach to improve the diagnosis of rare diseases [6]. Defining the genetic causes of DSDs by genome sequencing can identify the molecular etiology for definitive diagnosis in up to 64% of cases [7]. This study investigated twenty-one 46XY DSD patients without uterus. The molecular etiology was analyzed by WES to gain a more accurate and comprehensive understanding of the genetic causes and enrich the gene mutation database of 46, XY female DSDs. Based on the genotype results, combined with clinical phenotypes of 46, XY DSD patients without uterus to differentiate the abnormalities and make a definitive diagnosis.

## Materials and methods

### Patients

The 46, XY DSD patients without the uterus from unrelated families were enrolled in this study from the Department of Obstetrics and Gynecology, Reproductive Endocrinology and Infertility Center of the Peking Union Medical College Hospital from 2022 to 2023. Inclusion criteria included a 46, XY karyotype, female gender, absence of Mullerian duct structures, and presence or absence of external genital abnormalities. The 46, XY karyotype refers to the sex of the chromosome and is determined by G-banding. The term “female gender” refers to the classification of individuals as female based on biological and/or cultural characteristics associated with femininity. Informed written consent was obtained from all participants or the parents if the patient was under 18 years old. The study protocol was reviewed and approved by the Peking Union Medical College Hospital Ethics Committee (No. JS-2510).

### Clinical evaluations

Comprehensive clinical data, including chief complaint, age of presentation, results of physical examination related to sexual development, and surgical information were collected from medical records. Height was documented using the same meter and vaginal length was measured using a thin probe, with each measured three times for an average. The external genitalia and secondary sexual characteristics were evaluated by an experienced clinician. Breast development and pubic or axillary hair were recorded according to the Tanner stage. Pelvic ultrasounds were determined by the same experienced operator. Sex hormone levels included serum follicle-stimulating hormone (FSH), luteinizing hormone (LH), testosterone (T), estradiol (E2), and progesterone (P) levels were measured by an automated Elecsys Immunoanalyzer (Beckmann) in the same laboratory. Clinical features of the patients were analyzed, and blood samples were collected for WES.

### Whole exome sequencing

Genomic DNA was extracted from all patient samples using the QIAamp DNA Blood Mini Kit (Qiagen, Germany). First, DNA was fragmented into about 200 bp by fragmentation enzymes. DNA fragments were hybridized and captured following the manufacturer’s protocol. Next, the libraries were quantified by qPCR. The exons and DNA adjacent to the splicing region(20 bp) of 20,099 genes were then captured and enriched by Roche KAPA HyperExome chip. Finally, the Illumina HiSeq2000 platform, with 150 bp pair-end sequencing mode was utilized to sequence the exon regions and adjacent intronic regions. Sequencing fragments were aligned to the UCSC hg19 (GRCh37) human reference genome using BWA, with duplications removed by Picard. Base quality scores were corrected for single nucleotide variants (SNVs) and insertions/deletions (INDELs) using GATK. ExomeDepth was employed to detect copy number variations at the exon level.

### Assessment of variants

Variants were annotated using the Variant Effect Predictor (VEP) software and filtered based on quality, population frequency, and predicted impact on protein function. Variants with a minor allele frequency (MAF) > 1% in public databases (ESP6500, 1000 Genomes Project, Genome Aggregation Database, Exome Aggregation Consortium) were excluded. Pathogenicity was predicted using in silico tools such as SIFT, PolyPhen-2, Provean, and mutation taster. Variant pathogenicity was assessed based on the American College of Medical Genetics and Genomics (ACMG) and Society of Molecular Pathology (AMP) guidelines for interpreting sequence variants. Variants were classified as pathogenic, likely pathogenic, variants of uncertain clinical significance, likely benign, and benign. Potentially pathogenic variants were confirmed by Sanger sequencing. Trio-WES was performed on available family members to confirm inheritance patterns. Orthologous AR proteins were obtained from the Ensemble Genome Browser and aligned using the Clustal Omega multiple sequence alignment tool (EMBL-EBI) to evaluate evolutionary conservation. The missense variants of uncertain significance, AR variant p.Gly689Ala, and DHX37 variant p.Lys77Thr were modeled based on the structure model of AR (PDB ID:1e3g) and DHX37 (AlphaFold ID: AF-Q8IY37-F1). To observe the effects of missense variants on the tertiary structure of the protein, structural analyses were performed using Pymol.

## Results

A total of twenty-one 46, XY DSD patients without uterus from unrelated families were enrolled in this study. Based on the genetic results, 1 case of Leydig cell dysplasia (LCH), 5 cases of complete17α-hydroxylase deficiency(17OHD), 2 cases of 5α-reductase II deficiency(5α-RD2), 10 cases of androgen insensitivity syndrome (AIS), and two cases of 46 XY partial gonadal dysgenesis (PGD), previously misdiagnosed as partial androgen insensitivity syndrome (PAIS) were identified. Besides, one patient without *AR* mutation was clinically diagnosed with complete androgen insensitivity syndrome (CAIS).

### Clinical features

**Fig. 1:**
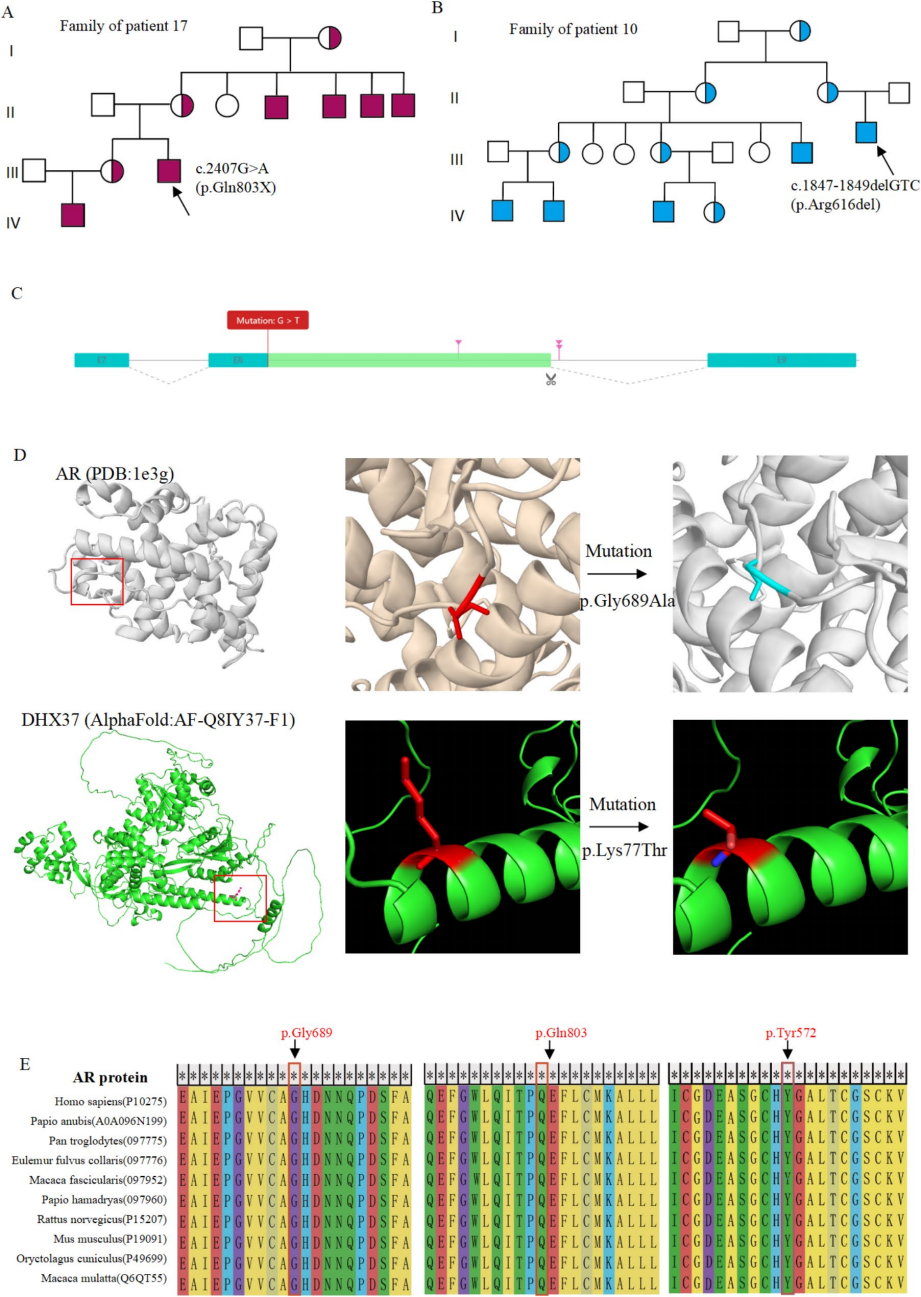
(**A, B**) Pedigree analysis of the patients’ families affected by complete androgen insensitivity. Women and men are represented by circles and squares, respectively. The black circles indicate the affected individuals and the semi-black circles indicate the carriers. The arrows indicate the patient of the cohort. (**C**) The effect of the splicing variant in *LHCGR*. (**D**) The effect of missense variants of AR and DHX37 on the tertiary structure of the protein. (**E**) Multiple sequence alignment analysis of the AR gene. Multiple amino acid alignment of AR including sequences from ten species. The mutant amino acids of the the *AR* gene and corresponding residues of aligned sequences are shown in red box

The clinical characteristics and hormone profiles of 21 patients are presented in Table 1. All patients were phenotypically female, and 46, XY chromosome karyotypes were confirmed in this cohort. The most common chief complaint among patients aged 13–27 years was primary amenorrhea (15 patients), and the remaining 6 preadolescent cases (Patients 6,7,8,13,20,21) presented with hypertension, the inguinal mass, or abnormal external genitalia. Genital examination revealed that some patients had normal female external genitalia while others presented with clitoral enlargement or bilateral large and small labia fused. Pelvic ultrasound and pathological findings confirmed the absence of a uterus in all patients. Excluding the 6 preadolescent cases, the height of the remaining patients ranged from 155 cm to 197 cm, with a median of 166.5 cm. In terms of the development of secondary sexual characteristics, all patients exhibited sparse or absent pubic hair. Patients with AIS exhibited relatively advanced breast development compared to those with LCH and 17OHD. Additionally, the five patients with 17OHD showed hypertension, and four of them were accompanied by hypokalemia. Based on the medical records, patients 10 and 17 had a family history as depicted in the family diagram (Fig. 1A and B). There were several hemizygous patients in four generations of the two families caused by the same variants.

**Table 1:** Clinical details of the individuals with variants causing 46, XY DSD without uterus

Pts	Gene mutations	Chief complaint	Age of presentation (years)	Height (cm)	Tanner stage	External genitalia	Pelvic ultrasound	Gonadectomy (location/pathology)	FSH (IU/l)	LH (IU/l)	E2 (pg/ml)	T (ng/ml)	*P* (ng/ml)
1	*LHCGR*	Primary amenorrhea	21	175	B2P1	Female, blind end of the vagina with 6 cm	The uterus not visible; bilateral inguinal nodules, cryptorchidism possible	Inguinal canal / Under-developed testes	8.31	35.05	< 15	0.56	0.39
**2**	CYP17A1	Primary amenorrhea	18	197	B2P1	Female, blind end of vagina with 4 cm	The uterus not visible; heterogeneous echo in the subendothelium of the left groin	Inguinal canal / Under-developed testes	41.8	55.92	< 15	< 0.1	14.04
3	CYP17A1	Primary amenorrhea	17	164	B1P1	Female, blind end of vagina with 5 cm	The uterus not visible; no mass in the bilateral groin	Position of the iliac vessels in the pelvic wall / Under-developed testes	148.66	53.90	< 15	0.17	8.97
4	CYP17A1	Primary amenorrhea	18	165	B1P1	Female, blind end of vagina with 5 cm	The uterus not visible; no mass in the bilateral groin	The internal opening of the inguinal canal /Juvenile tubular gonadal tissue	93.5	56.58	< 15	< 0.1	13.10
5	CYP17A1	Primary amenorrhea	23	163	B1P1	Female, blind end of vagina with 5 cm	The uterus not visible; hypoecho in the left groin	The inguinal canal / Under-developed testes	55.35	16.35	< 15	0.22	12.08
6	CYP17A1	Hypertension	12	155	B1P1	Female, blind end of vagina with 4 cm	The uterus not visible; no mass in the bilateral groin	-	54.53	14.4	< 15	0.11	3.4
**7**	SRD5A2	Inguinal mass	At birth	155	B1P1	Female, elevated perineal body	The uterus not visible; abnormal echo in the bilateral groin	-	6.26	3.66	< 15	0.61	0.26
8	SRD5A2	External genital abnormalities	At birth	110	B1P1	Bilateral large and small labia fused, elevated perineal body, resembling a vaginal opening, clitoromegaly	The uterus not visible; solid nodule in the groin	-	-	-	-	-	-
9	AR	Primary amenorrhea	27	165	B4P1	Juvenile female; blind end of vagina with 6 cm	The uterus not visible; no mass in the groin;	The pelvic wall near the anterior superior iliac spine /Under-developed testes	28.63	35.8	< 15	0.34	1.11
10	AR	Primary amenorrhea	18	176	B2P1	Juvenile female, blind end of vagina with 7 cm	The uterus not visible; mass in the bilateral inguinal region	The groin / Under-developed testes	65.53	32.33	< 15	0.63	0.27
11	AR	Primary amenorrhea	21	168	B3P1	Juvenile female, blind end of vagina with 3 cm; Inguinal hernia at 3year	The uterus not visible;	The groin / Under-developed testes	66.89	22.36	< 15	0.48	0.67
12	AR	Primary amenorrhea	16	175	B5P1	Juvenile female; blind end of vagina with 4 cm;	The uterus not visible;	The inguinal canal / Under-developed testes	3.56	16.30	20	5.24	0.23
13	AR	External genital abnormalities	2	-	-	2 cm in diameter, mobile nodules bilaterally within labia majora; 1 × 0.6 cm clitoris	The uterus not visible; Bilateral inguinal hypoecho, cryptorchidism possible	-	10.81	0.82	6	0.025	0.05
14	AR	Primary amenorrhea	13	155	B2P1	Juvenile female, blind end of vagina with 2 cm	The uterus not visible; the right iliac vessels 3.2*1.0 cm hypoecho	-	7.11	13.81	18	2.70	0.24
15	AR	Primary amenorrhea	14	172	B3P1	Juvenile female, blind end of vagina with 2 cm	The uterus not visible; solid echo on the pelvic iliac vessels	The pelvic wall near the anterior superior iliac spine / Under-developed testes	43.13	45.39	21	3.46	0.51
16	AR	Primary amenorrhea	13	158	B2P1	Juvenile female, blind end of vagina with 4 cm; Inguinal hernia at 2year	The uterus not visible; solid nodules in the bilateral iliac fossa	-	12.34	27.78	< 15	3.15	0.28
17	AR	Primary amenorrhea	15	156	B3P1	Juvenile female, blind end of vagina with 4 cm	The uterus not visible; testicular echoes in both inguinal regions	The groin / Under-developed testes	32.51	41.72	24	6.23	0.38
18	AR	Primary amenorrhea	22	163	B5P1	Juvenile female, blind end of vagina with 6 cm	The uterus not visible; solid nodules in the bilateral iliac fossa	Within iliac fossa / Under-developed testes	10.04	38.34	65	5.32	0.76
19	DHX37	Primary amenorrhea	15	175	B5P1	Juvenile female, blind end of vagina with 4 cm	The uterus not visible; bilateral external iliac vessels medial soft tissue structures, gonads possible	The internal orifice of the groin / Under-developed testes	7.70	20.17	23	5.01	< 0.08
20	NR5A1	External genital abnormalities	5	170	B1P1	Clitoris hypertrophy with 4*1*1 cm, Prader grade 3, 2*2*1.5 cm nodules palpated within the labia major bilaterally	The uterus not visible	Labium majus/ Epididymis, testis and spermatic cord tissue, consistent with gonadal dysplasia	73.99	13.62	17	3.01	0.32
21	NR5A1	External genital abnormalities	10	163	B1P1	Clitoris hypertrophy with 5*2*2 cm	The uterus not visible; solid nodules in the grion	inguinal canal/ consistent with gonadal dysplasia	51.63	30.09	< 15	3.36	0.19

Note: The reference ranges of the sex hormone for adult males: FSH:1.27-12.96IU/L, LH:1.24-8.62IU/L, E2:20-75pg/ml, P:0.1-0.84ng/ml, T:1.75-7.81ng/ml. The reference ranges for female sexual hormone of follicular stage are as follows: LH 2.12–10.89 IU/l; FSH < 10IU/l; T 0.10–0.84 ng/ml; E2 22-115pg/ml; P 0.38-2.28ng/ml. -: not available

*Tanner grade: B:breast, P:pubic hair, divided into five grades

### Characters of hormones

Hormonal findings in prepubertal children (Patients 7, 13) were age-appropriate due to the inactive hypothalamic-pituitary-gonadal axis. Except for the patients with AIS and 46XY PGD, the remaining patients showed significantly reduced androgen levels, ranging from < 0.1ng/ml to 0.61ng/ml, due to insufficient synthesis and reduction. Patients with 17α-hydroxylase deficiency are characterized by persistent hyperprogestinemia(P range: 3.4-14.04 ng/mL), accompanied by decreases in E2 (< 15pg/ml) and T, and increases in FSH (41.8-148.66IU/L) and LH (14.4-56.58 IU/L).In patients with AIS, E2 levels ranged between 6pg/mL and 65pg/mL; P levels were confirmed with preovulation status; six cases (patients9, 10,11, 15,17 and 19) showed FSH levels greater than 20 IU/L. Notably, three cases (10, 11, and 15) presented with FSH levels above 40 IU/L. LH levels > 20 IU/L were observed in 7cases(patients 9, 10, 11, 15,16, 17, and 18). The T levels were consistent with or higher than normal men, except in three cases (9, 10, and 11), where T levels were below 0.84 ng/ml. Two patients with 46, XY partial gonadal dysgenesis presented with hypergonadotropic hypogonadism, as evidenced by FSH (51.63–73.99 IU/L), accompanied by elevated T levels (3.01–3.36 ng/mL).

### Mutational analysis

WES identified 22 variants in the genes associated with sex development as illustrated in Table 2, which include 14 recurrent variants and 8 novel variants in the genes involved in steroid hormone synthesis and androgen receptor action (*LH/HCG receptor (LHCGR)* (2/22), *CYP17A1* (4/22), *SRD5A2* (3/22), the *AR* (10/22)), and the gene associated with gonadal development *(NR5A1*(2/22)). Additionally, a DHX37 variant instead of an AR variant was detected in patient 19 whose clinical manifestations are consistent with CAIS. Based on the ACMG guidelines, 17 variants were classified as pathogenic(P) or likely pathogenic (LP) variants and 5 variants were defined as variants of uncertain significance (VUS). Trio-WES showed there are three de novo variants. The inheritance patterns of each disease are described in Table 2.

**Table 2:** The detailed information of variants detected in patients with 46, XY DSD without uterus

Patient	Gene	Base change	Amino acid change	Location	Previously published	dbSNP	Clinvar (Variation ID)	ACMG classification	Inheritance	Inheritance pattern
1	LHCGR	EX1-EX6 Del	-	EX1-EX6	No	-	-	P	Maternal	AR
		c.680 + 1G > T	-	IVS8	No	-	-	VUS	De novo	
2	CYP17A1	c985-987del TACinsAA	p.Tyr329Lysfs*90	EX6	Yes	rs1844105842	Pathogenic​(969804)	P	De novo	AR
3	CYP17A1	c.985-987delT ACinsAA	p.Tyr 329Lysfs*90	EX6	Yes	rs1844105842	Pathogenic​(969804)	P	Maternal	AR
		c.161-173delT CAAGCTGCA GAA	p.Phe54*	EX1	No	-	-	P	Paternal	
4	CYP17A1	c.985-987delT ACinsAA	p.Tyr 329Lysfs*90	EX6	Yes	rs1844105842	Pathogenic​(969804)	P	Maternal	AR
		c.1084 C > T	p.Arg362Cys	EX6	Yes	rs104894142	Pathogenic(1796)	P	Paternal	
5	CYP17A1	c.985-987delT ACinsAA	p.Tyr329Lysfs*90	EX6	Yes	rs1844105842	Pathogenic​(969804)	P	Maternal	AR
		c.316T > C	p.Ser106Pro	EX2	Yes	rs104894135	Pathogenic​(1780)	LP	Paternal	
6	CYP17A1	c.985-987delT ACinsAA	p.Tyr 329Lysfs*90	EX6	Yes	rs1844105842	Pathogenic​(969804)	P	Maternal/ Paternal	AR
7	SRD5A2	c.16 C > T	p.Gln 6*	EX1	Yes	rs9332960	Pathogenic​(436859)	P	Maternal	AR
		c.680G > A	p.Arg227Gln	EX5	Yes	rs9332964	Likely pathogenic​(3351)	P	Paternal	
8	SRD5A2	c.680G > A	p.Arg227Gln	EX5	Yes	rs9332964	Pathogenic/Likely pathogenic​(3351)	P	Maternal	AR
		c.607G > A	p.Gly203Ser	EX5	Yes	rs9332961	pathogenic​(459640)	P	Paternal	
9	AR	c.2678 C > T	p.Pro893Leu	EX8	Yes	rs1602280356	Pathogenic/Likely pathogenic (654813)	LP	Maternal	XR
10	AR	c.1847-1849delGTC	p.Arg616del	EX3	Yes	-	-	LP	Maternal	XR
11	AR	c.2066G > C	p.Gly689Ala	EX4	No	-	-	VUS	Maternal	XR
12	AR	c.2301del	P.Asp768Ilefs*21	EX5	Yes	rs886041131	Pathogenic(279686)	P	Maternal	XR
13	AR	c.1720G > C	p.Ala574Pro	EX2	Yes	rs1057521121	Likely pathogenic​(381643)	VUS	Maternal	XR
14	AR	c.2495G > A	p.Arg832Gln	EX7	Yes	rs1386577803	Pathogenic​( 458366)	P	Maternal	XR
15	AR	c.2324G > A	p.Arg775His	EX6	Yes	rs137852572	Pathogenic​​( 9819)	P	Maternal	XR
16	AR	c.1736G > T	p.Ser579Ile	EX2	Yes	-	-	LP	Maternal	XR
17	AR	c.2407 C > T	p.Gln803*	EX6	No	-	-	LP	Maternal	XR
18	AR	c.1716T > G	p.Tyr572*	EX2	No	-	-	LP	Maternal	XR
19	DHX37	c.230 A > C	p.Lys77Thr	EX2	No	-	-	VUS	Maternal	AD
20	NR5A1	c.251G > A	p.Arg84His	EX4	Yes	rs375469069	Pathogenic​​( 641278)	P	Maternal	AD
21	NR5A1	c.1363-1371del9	p.Met455-Gln457del	EX7	No	-	-	VUS	De novo	AD

Abbreviation: Inheritance pattern: AR: autosomal recessive inheritance; XR: X-linked recessive inheritance; AD: autosomal dominant inheritance

### Analysis of the novel variants identified in patients

We evaluated the novel variants by in-silico analysis. The study identified two novel variants in the LHCGR gene: a large deletion from EX1 to EX6 and an intronic splicing variant (c.680 + 1G > T). The splice variant was predicted in slico (https://rddc.tsinghua-gd.org/ai/rna-splice), suggesting that the original splice recognition site was disrupted, leading to the use of a potential alternative splice site in the intron and the inclusion of part of the intronic sequence (Fig. 1C). The frameshift mutation caused premature termination. The other novel variants were also predicted to be deleterious by four prediction tools, including Polyphen-2, SIFT, Provean, and Mutation Taster (Table 3). We further explored the tertiary structure of the proteins with two missense variants defined as VUS. As shown in Fig. 1D, they affected the tertiary structure of the proteins, and p. Gly689Ala caused disulfide breakage. Multiple sequence alignment analysis was performed to analyze the evolutionary conservancy across different species. Our results showed that the three variants in AR, p.Gly689Ala, p.Gln803*, and p.Tyr572*, all affected the strictly conserved domains among vertebrate orthologs, including ten distantly related species (Fig. 1E). Sequences with longer bases are shown in Supplementary Fig. 1.

**Table 3:** In Silico analysis of novel variants found by WES

Gene	Base change	Mutation type	PolyPhen/ SIFT/ Provean/ MutationTaster	ACMG tags	ACMG classification
LHCGR	EX1-EX6 Del	Deletion	-/-/-/-	PVS1 + PM2 + PP4(LOF)	P
	c.680 + 1G > T	splice-site	-/-/-/D	PVS1_Moderate + PM2 + PP4	VUS
CYP17A1	c.161-173delTCAA GCTGCA GAA	Frameshift	-/-/-/D	PVS1 + PM2 + PM3_Supporting	P
AR	c.2066G > C	Missense	D/D/D/D	PM2 + PP2 + PP3	VUS
AR	c.2407 C > T	Nonsense	-/-/-/D	PVS1 + PM2_Supporting	LP
AR	c.1716T > G	Nonsense	-/-/-/D	PVS1 + PM2_Supporting	LP
DHX37	c.230 A > C	Missense	B/D/D/N	PM2 + PP2	VUS
NR5A1	c.1363-1371del9	Deletion	-/-/-/D	PM2_Supporting + PM4 + PM6	VUS

Abbreviation: **PolyPhen**http://genetics.bwh.harvard.edu/pph2/. D: Probably damaging ( > = 0.957), P: possibly damaging (0.453 < = pp2_hdiv < = 0.956) B: benign(pp2_hdiv < = 0.452), **SIFT**/**Provean**http://provean.jcvi.org/protein_batch_submit.php?species=human. D: Damaging (sift < = 0.05); T: tolerated (sift > 0.05)/ Deleterious(-14 < provean<-2.5); T: tolerated (-2.5 < provean < 14), **MutationTaster**http://www.mutationtaster.org/. A: disease causing_automatic, D: disease_causing, N: polymorphism, P: polymorphism_automatic

## Discussion

The 46, XY DSDs can be further subdivided into three main categories: gonadal dysgenesis due to gonadal development problems, insufficient androgen production (testosterone and dihydrotestosterone) due to defects in biosynthesis, or target organ resistance to androgen due to defects in androgen receptor [8]. During sexual differentiation, leydig cells respond to the LH/HCG stimulation producing testosterone and activating AR, which works with AMH to complete internal genital differentiation. Testosterone is catalyzed by SRD5A2 to produce DHT, which activates AR to complete male external genital differentiation. Mutations in genes related to steroid biosynthesis (such as CYP11A1, CYP17A1, HSD3B2, HSD17B3, and StAR) will lead to androgen synthesis disorders with adrenal insufficiency besides the SRD5A2 gene mutation which results in the insufficient transformation of dihydrotestosterone from testosterone [1]. The mutation of *AR* gene will lead to resistance to the biological action of androgens, despite normal androgen concentrations in males [9]. LCH, 17OHD, 5α-RD2, and AIS result from disorders of androgen synthesis and action during sexual differentiation. 46, XY PGD arises from partial impairment of testicular function due to obstruction of testicular formation during the testicular determination stages.

Traditionally, the diagnosis of 46, XY DSDs relies on detailed medical history, comprehensive clinical evaluation, and related examinations including chromosome karyotype and hormone measurement [10–11]. However, due to the limitations of biochemical tests and imaging examinations, as well as the similarity of clinical phenotypes, distinguishing the etiology remains challenging in some cases, primarily due to the varying degrees of virilization caused by partial androgen effects [12]. Advancements in genomics over the last decade have significantly enhanced our comprehension of the genetic basis of DSDs, with genome sequencing technology emerging as a primary diagnostic tool. Precision medicine uses modern genetics and bioinformatics technology to determine patients’ genetic background and disease characteristics. As an important tool in precision medicine, reverse phenotyping predicts phenotypes from genotypes, to achieve accurate classification and molecular etiology diagnosis of diseases [13]. This study identified the molecular etiology of 21 cases of 46, XY DSD patients without uterus in 2 years of our hospital based on WES, combined with the clinical phenotypes, revealing one patient with LCH, five patients with 17OHD, two patients with 5α-RD2, and eleven patients with AIS. What’s more, two patients with misdiagnosed PAIS were found to have 46, XY PGD(OMIM:617480). This further emphasizes the importance of reversing phenotyping based on genetic results.

An important finding in this study is variants in *NR5A1*, which correct the misdiagnosis of PAIS in two patients with 46, XY partial gonadal dysgenesis (OMIM:617480). 46, XY PGD is characterized by partial testis differentiation. External genital virilization degrees vary based on the amount of functional testicular tissue present in the individual’s gonad [14]. NR5A1 mutations are linked to a broad range of gonadal development disorders, spanning from DSD to oligo/azoospermia in 46XY individuals and 46XX ovotesticular and testicular phenotypes to primary ovarian failure in 46XX individuals. Studies had indicated that polygenic inheritance or pathogenic variants in other testis/ovarian-determining gene might account for the extensive phenotypic variability associated with NR5A1 gene mutations [15]. Here, we identified two patients carrying the heterozygous NR5A1 variant (p.Arg84His and p.Met455-Gln457del), who presented with partial virilization and absence of Mullerian duct structures, overlapping with the phenotype of PAIS, which led to our misdiagnosis. The variant of p.Arg84His has been reported. It can be inherited from an asymptomatic mother, with functional studies indicating its potential to cause impaired NR5A1 protein cytosolic retention and transcriptional activation [16]. The novel deletion variant p.Met455-Gln457del was assessed as a variant of uncertain significance, which was predicted to be deleterious in slico. Previous studies have observed the absence of Mullerian duct structures in 46, XY partial gonadal dysgenesis, suggesting that certain *NR5A1* genetic variants may exert a more significant impact on the steroidogenic pathway than on Sertoli cell function [17]. Sertoli cell function appears to be preserved in the fetus, playing a role until Mullerian ducts degenerate [18]. In our research, the two patients had a female genital assessment at birth, yet masculinizing manifestations such as clitoral enlargement occurred with the onset of pubertal development. This spontaneous virilization phenomenon suggests that NR5A1 may have a lesser role in steroidogenesis during puberty than during fetal life [19]. Research on a hypomorphic mouse model of NR5A1 revealed differential impairment of fetal and adult leydig cell development, whereby NR5A1 may regulate the differentiation of fetal leydig cells, whereas in the adult it may regulate progenitor cell formation and/or survival [20]. Different actions of NR5A1 in fetal and postnatal leydig cell populations might contribute to the switch from birth to pubertal phenotypes.

Numerous cohort studies worldwide focus on 46, XY DSDs, with genetic spectrums varying among populations, though pathogenic variants in *AR* are the most prevalent genetic etiology. Studies have shown that over 1100 different *AR* pathogenic variants have been registered in the database species, including deletions, duplications, insertions, missense (the most common), and nonsense variants [21].Here, we identified ten patients carrying *AR* variant and one patient carrying *DHX37* variant, combined with clinical phenotype, consistent with the diagnosis of AIS(OMIM:312300), which included 1 patient with PAIS presented with ambiguous genitalia and 10 patients with CAIS presented with complete female phenotype. Three novel *AR* variants (p. Gly689Ala, p. Gln803Ter and p. Tyr572*) were identified, which were predicted to be deleterious in slico. The p.Gly689Ala variant defined as VUS by ACMG, affects the disulfide bond, considered a candidate for AIS combined with the clinical manifestation, but requires further studies to establish pathogenicity. Multiple sequence alignment analysis demonstrated they impacted highly strictly conserved domains among vertebrate orthologs, spanning ten species (Fig. 1E).The family history of CAIS in patient 10 and patient 17 merits attention. Despite the ACMG defining variants p.Arg616del and p.Gln803Ter as LP, their family history suggests their pathogenicity. The variants were inherited from their healthy mothers consistent with the X recessive inheritance pattern.

It is perplexing that the patient 19’s clinical manifestations align with CAIS (female with juvenile external genitalia, blind end of vagina, testis located in inguinal region, admitted with primary amenorrhea), yet no mutation of *AR* gene was detected. Instead, a novel *DHX37* variant involved in gonadal development was detected [22, 23]. Other mechanisms by which androgens may fail to act are conceivable and warrant exploration through additional functional testing. The occurrence of CAIS cases without *AR* mutations is not uncommon, as previously reported [24]. Studies have reported that approximately 10% of CAIS and 60–80% of PAIS patients lack such mutations [25]. Studies of such patients described mutations in other genes crucial for sexual development, such as the hypospadias-associated *MAMLD1* gene [26] or the *FKBP4* gene, encoding a regulator of the AR signaling pathway [27]. The mutated *DHX37*, potentially serving as a coregulatory protein, might influence the AR signaling pathway, but this hypothesis necessitates further detailed analysis.

*CYP17A1* was the second frequently identified variant in our cohort. Two homozygous and three compound heterozygous variants were found in the five patients with complete 17OHD (OMIM:2021100). Over 100 different CYP17A1 gene variants have been reported, encompassing missense, small insertions or deletions, and splice site variants (http://www.hgmd.cf.ac.uk). Four P/LP variants were identified in the study, including a novel frameshift mutation p.Phe54* in exon 1. The frame-shift variant c.985_987delTACinsAA (p.Tyr329fs) identified in every patient reported as a common variant in the Chinese population in exon 6 [28]. Besides, we identified two patients carrying the complex heterozygous variants of *SRD5A2*, who presented with varying degrees of ambiguous external genitalia, diagnosed as 5α-RD2(OMIM:264600). Both variants were inherited from their healthy parents. PGln6* was reported to be a hotspot variant, detected in half of SRD5A2 variant patients. This variant results in a truncated protein lacking binding sites for testosterone and cofactors, associated with severe phenotype and lower external masculinization score and urethral meatus score, as reported [29]. In our study, patient 7 with this variant exhibited a more severe undervirilized phenotype than patient 8, consistent with this finding. A patient with LCH (OMIM:238320) was identified based on the genetic results, carried a large deletion from EX1 to EX6 in the LHCGR gene, and an intronic splicing variant (c.680 + 1G > T), which is assessed as P (PVS1 + PM2 + PP4) and VUS (PVS1_Moderate + PM2 + PP4) by ACMG, respectively. The splicing variant is predicted to affect gene splicing, resulting in a frameshift mutation. Cases of LCH are relatively rare, with over 20 LHCGR inactivating mutations identified so far, scattered throughout the gene [30]. However, most patients with the clinical phenotype of LCH lack pathogenic mutations, suggesting that alterations in other regions of the *LHCGR* gene, such as the large intron or promoter region, may account for the majority of cases [31].

There are a few limitations in the study. Firstly, due to the limited number of samples and the fact that most of the included patients were 46 XY DSDs with relatively clear etiology of androgen synthesis or action disorders, the diagnostic rate in the cohort was 100%. Although the implementation of next-generation sequencing methods has greatly improved the diagnostic rate of DSD patients with unknown etiology, half of the cases still have unknown genetic causes, which may be related to epigenetics or potential noncoding variants [32]. Epigenetic analysis was not involved in this study, and further studies with larger sample sizes are needed. Secondly, the deleteriousness of the variants was only predicted in slico, and has not been validated in vitro and in vivo, requiring further functional studies.

## Conclusion

Here, we facilitate the molecular diagnosis in 46, XY DSD patients without uterus using reverse phenotyping and identified several novel variants broadening the mutation spectrum of these diseases. The pathogenesis of 46XY DSDs without uterus is complex, involving disorders of both androgen synthesis and AR action in the stage of sex differentiation, as well as 46XY partial gonadal dysgenesis in the stage of gonadal development. Reverse phenotyping based on the genotype results combined with clinical phenotypes helps differentiate the abnormalities, uncover the underlying causes of certain rare diseases, and make a definitive molecular diagnosis, which can not only prevent the occurrence of malignant tumors but also reduce the birth of affected children.

## Electronic supplementary material

Below is the link to the electronic supplementary material.

Supplementary Material 1

## Data Availability

The datasets used and/or analyzed during the current study are also available from the corresponding author on reasonable request.

## Abbreviations

DSD: Disorders of sex development
WES: Whole exome sequencing
P: Pathogenic
LP: Likely pathogenic
VUS: Variants of uncertain significance
US: Ultrasound
FSH: Follicle-stimulating hormone
LH: Luteinizing hormone
T: Testosterone
E2: Estradiol
P: Progesterone
ACMG: American College of Medical Genetics and Genomics
LCH: Leydig cell dysplasia
17OHD: 17α-hydroxylase deficiency
5α-RD2: 5α-reductase II deficiency
AIS: Androgen insensitivity syndrome
CAIS: Complete androgen insensitivity syndrome
PAIS: Partial androgen insensitivity syndrome
46, XY PGD: 46, XY partial gonadal dysgenesis
