# Supplementary figures and images for: The clinical diversity and molecular etiology in 46, XY disorders of sex development patients without uterus
Source: Orphanet J Rare Dis. 2025 Apr 17;20:187. doi: 10.1186/s13023-025-03719-y (PMC12007265; doi:10.1186/s13023-025-03719-y)

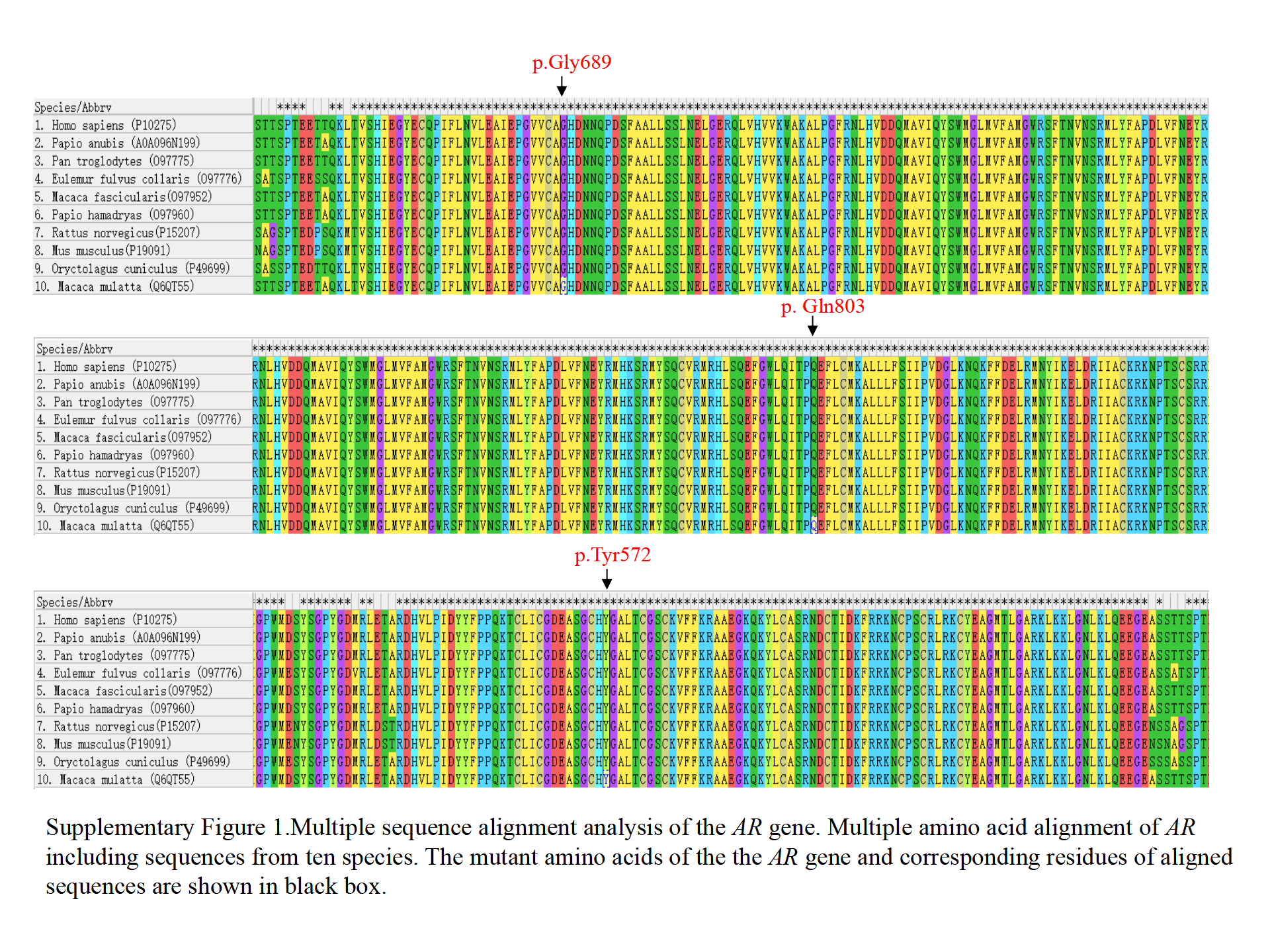

Supplement: Supplementary file 1 — Supplementary Material 1 [file 13023_2025_3719_MOESM1_ESM.png]
